# Surrogate Endpoints in CLL: From Promise and Pitfalls to a Context-Specific Validation Framework

**DOI:** 10.3390/hematolrep18040050

**Published:** 2026-07-21

**Authors:** Stefano Molica

**Affiliations:** Department Haematology, Castle Hill Hospital, Hull-York Medical School, Cottingham HU16 5JQ, UK; stefano.molica@hyms.ac.uk or smolica@libero.it

**Keywords:** CLL, surrogate endpoints, TTNT, MRD, QoL

## Abstract

Surrogate endpoints are increasingly used in chronic lymphocytic leukemia (CLL) to accelerate treatment evaluation, but their validity is context-dependent. This review examines key endpoints—progression-free survival (PFS), time to next treatment (TTNT), measurable residual disease (MRD), and quality of life (QoL)/patient-reported outcomes (PROs). PFS, while standard, is limited by competing risks and poor reflection of toxicity and patient experience. TTNT captures both efficacy and tolerability but is influenced by external factors. MRD is a strong predictor of outcomes in fixed-duration venetoclax-based regimens but less reliable in continuous therapies. QoL and PROs provide essential patient-centered insight often missed by traditional endpoints. We propose a practical framework in which endpoint selection depends on treatment type, patient characteristics, and intended use. MRD is most informative after fixed-duration therapy, TTNT in continuous treatment, and PROs in vulnerable populations. Overall, surrogate endpoints in CLL require setting-specific validation to ensure they reflect meaningful clinical benefit.

## 1. Introduction

Chronic lymphocytic leukemia (CLL) is characterized by a prolonged and heterogeneous clinical course, in which survival may extend over years or decades and treatment is often administered across multiple lines [1]. Over the past decade, the rapid expansion of targeted therapies—including Bruton tyrosine kinase inhibitors (BTKis) and BCL2 inhibitors—has profoundly reshaped disease trajectories, shifting CLL from a chemoimmunotherapy-based paradigm to one defined by continuous oral therapies and time-limited, highly effective combinations [1]. These changes resulted in significant improvements in survival outcomes for patients with CLL across treatment eras, with the greatest gains observed during the modern targeted therapy era [2].

While these advances have improved patient outcomes, they have also complicated the assessment of clinical benefit, as traditional endpoints such as overall survival (OS) require long follow-up and may be confounded by subsequent therapies [2,3].

In this evolving context, surrogate endpoints have gained increasing importance as tools to accelerate clinical research and regulatory decision-making [2]. Endpoints such as progression-free survival (PFS), time to next treatment (TTNT), and measurable residual disease (MRD) enable earlier evaluation of therapeutic efficacy, reducing trial duration, cost, and complexity while facilitating timely access to novel agents [3,4,5,6]. However, in CLL, the validity of surrogate endpoints is not universal. Rather, their interpretability is closely linked to treatment mechanism, duration of therapy, and patient characteristics [4]. A surrogate that correlates with outcomes in one therapeutic setting may not retain its predictive value in another [4]. This is exemplified by MRD, which shows a strong association with PFS in fixed-duration venetoclax-based regimens but lacks consistent correlation in the setting of continuous BTKi therapy, where durable clinical benefit may occur despite persistent detectable disease [5,7].

Importantly, the concept of clinical benefit in CLL extends beyond disease control alone. The 2026 European Hematology Association (EHA) CLL guidelines assessed the Magnitude of Clinical Benefit Scale (ESMO-MCBS) for the main frontline therapies in CLL, providing a structured framework to compare highly effective treatment options based not only on efficacy but also on toxicity, treatment duration, and monitoring requirements [8]. This represents an important step toward a patient-centered approach to CLL management, placing patients’ preferences, quality of life, and treatment burden alongside clinical efficacy in therapeutic decision-making.

Given the chronic nature of the disease and prolonged treatment exposure—particularly with continuous therapies—treatment-related toxicities, quality of life (QoL), and patient-reported outcomes (PROs) represent critical dimensions of outcome assessment. Surrogate endpoints that fail to capture these elements risk providing an incomplete estimate of net clinical benefit, particularly in older and comorbid populations [9].

These considerations underscore a key challenge: surrogate endpoint selection in CLL should not be viewed as a purely methodological exercise, but as a context-dependent decision integrating biological, therapeutic, and patient-centered factors [10]. In this review, we explore the strengths and limitations of commonly used surrogate endpoints in CLL and propose a pragmatic framework to guide their interpretation and application within the continuously evolving therapeutic landscape.

## 2. Literature Search and Study Selection

This narrative review was conducted using a structured literature search approach. PubMed/MEDLINE, Embase, and major hematology conference proceedings (ASH, EHA) were searched for studies published between January 2010 and January 2026. Search terms included combinations of “chronic lymphocytic leukemia,” “surrogate endpoints,” “progression-free survival,” “time to next treatment,” “minimal residual disease,” “quality of life,” and “patient-reported outcomes.”

Eligible studies included randomized controlled trials, prospective studies, meta-analyses, and relevant regulatory or methodological papers addressing endpoint validation in CLL. Conference abstracts and in-press articles were included when highly relevant to emerging therapeutic strategies.

Studies were selected based on relevance to endpoint interpretation, methodological rigor, and representation of different therapeutic classes (BTK inhibitors, BCL2 inhibitors, and combination regimens). Data extraction focused on endpoint definitions, strengths, limitations, and reported associations with clinical outcomes. Given the narrative nature of the review, no formal systematic review or meta-analysis methods were applied.

**Figure 1:**
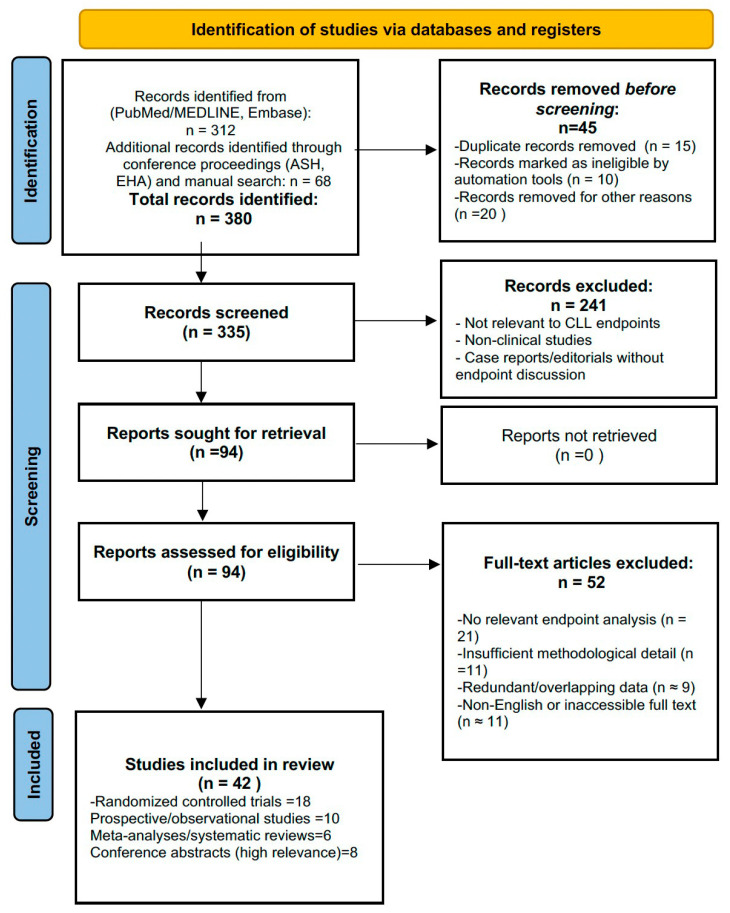
Preferred Reporting Items for Systematic reviews and Meta-Analyses (PRISMA) flow diagram for the systematic review and meta-analysis.

The analysis adhered to PRISMA guidelines (Figure 1). Evidence was interpreted according to a hierarchy prioritizing randomized trials and meta-analyses, followed by prospective studies, with preliminary findings clearly identified as such.

## 3. PFS, a Sometimes-Misleading Endpoint?

PFS, defined as the time from the start of therapy to objective disease progression or death from any cause, serves as a widely adopted primary endpoint in CLL clinical trials [4]. The composite nature of PFS allows to capture key aspects of treatment efficacy, including favorable disease biology, tumor burden reduction, and delayed relapse, often correlating with OS [4]. However, several inherent limitations undermine the interpretability and comparability of PFS in the modern CLL landscape, dominated by targeted therapies, and evolving assessment standards [4,6].

A primary caveat arises from competing risks in frail patient cohorts. In older CLL patients with substantial comorbidities PFS conflates CLL progression with non-disease-related deaths, obscuring a treatment’s specific antitumor activity and complicating cross-trial comparisons [6]. Many deaths occur without documented progression, diluting PFS as a pure measure of disease control. The phase III CLL14 trial exemplifies this issue: in untreated patients with comorbidities (Cumulative Illness Rating Scale [CIRS] score > 6 or creatinine clearance < 70 mL/min), fixed-duration venetoclax plus obinutuzumab (VO) was compared to chlorambucil plus obinutuzumab (CO). At a median follow-up of 71.9 months, 35% of PFS events in the VO arm were deaths without prior progression, versus 48% in the CO arm, highlighting how competing mortality distorts PFS estimates and challenges its surrogacy for OS [11].

PFS is highly sensitive to imaging frequency assessment, response criteria (e.g., iwCLL 2018), and investigator discretional evaluation, introducing variability across trials [12,13]. The phase III BRUIN CLL-321 trial underscores this point. In such a trial, pirtobrutinib, a non-covalent BTKi, was compared to bendamustine plus rituximab (BR) or idelalisib plus rituximab in relapsed/refractory CLL. Notably, 62% of progression events in the pirtobrutinib arm were asymptomatic, detected via scheduled computed tomography (CT) scans rather than symptomatic decline, contrasting with the control arms where progressions were more symptom-driven. Moreover, approximately 38% of pirtobrutinib-treated patients continued therapy beyond Independent Review Committee (IRC)-confirmed progression due to clinical benefit, as permitted by protocol. Post-progression continuation of pirtobrutinib may weaken the association between PFS and clinically meaningful patient outcomes, thereby potentially diminishing the predictive validity of PFS [14].

Beyond these pitfalls in the efficacy assessment, PFS fails to encapsulate holistic treatment effects. It does not inherently account for long-term toxicities, such as cumulative infections, cardiovascular events, or secondary malignancies associated with continuous BTKi or BCL2 inhibitor regimens, nor does it capture QoL tra-jectories via PROs [15].

In summary, while PFS remains a cornerstone endpoint in CLL trials due to its sensitivity to early efficacy signals, its interpretation demands contextualization. Future trial designs should integrate toxicity-adjusted composites (e.g., event-free survival excluding non-CLL deaths), standardized PRO assessments, and real-world evidence to mitigate these limitations, ensuring PFS better reflects net clinical benefit in an era of paradigm-shifting therapies [15].

## 4. Time to Next Treatment (TTNT), a Pragmatic Endpoint in CLL

TTNT has emerged as a complementary endpoint that measures the interval from treatment initiation to the start of subsequent therapy [4,6]. Unlike PFS, TTNT incorporates treatment efficacy together with tolerability, adherence, quality of life, and physician decision-making, making it a pragmatic indicator of real-world clinical benefit [4,6].

The interpretive value of TTNT is critically dependent on the treatment regimen duration—fixed versus continuous—which differentially shapes its sensitivity to efficacy and safety signals [6]. In fixed-duration regimens, TTNT primarily reflects efficacy and tolerability during the planned treatment period; following treatment discontinuation, it predominantly captures the durability of disease control, with limited confounding from ongoing treatment-related toxicity [13,16,17,18,19,20,21]. In contrast, in continuous therapy, TTNT integrates both efficacy and tolerability across the entire treatment course, as treatment is maintained until disease progression or unacceptable toxicity [22,23,24,25]. Consequently, TTNT is continuously influenced by sustained disease control as well as the cumulative burden of treatment-related adverse events over time [6].

In BTKi-based regimens, cardiovascular adverse events (e.g., atrial fibrillation) and infections, which may in some cases precede disease progression, can significantly contribute to treatment discontinuation and, consequently, shorten time to TTNT [26]. Specifically, in the ELEVATE-TN trial evaluating acalabrutinib with or without obinutuzumab versus obinutuzumab–chlorambucil, discontinuations due to adverse events (22%) underscore treatment-related toxicity as an important determinant shortening TTNT beyond what would be expected based solely on disease progression dynamics [22].

A recent analysis of 13 prospective randomized trials in CLL, including both continuous and fixed-duration targeted therapies, found strong trial-level concordance between PFS and TTNT. These findings further support TTNT as a surrogate for PFS [6].

However, results may be biased by different follow-up durations, censoring patterns, and a diversity of subsequent therapies, all of which can complicate the interpretation of the TTNT–PFS relationship. Regulatory and methodological perspectives advise caution when considering TTNT as a proxy for PFS [27,28]. Across European health technology assessment bodies (e.g., EUnetHTA) and U.S. regulators, such as the FDA, a two-level validation framework is required for surrogate endpoint approval [27,28]. This framework emphasizes the need to establish meaningful patient-level associations and to corroborate trial-level surrogacy before any surrogate endpoint can inform decision-making, while avoiding overinterpretation of TTNT’s role [15].

TTNT’s utility is tempered also by susceptibility to extra-biological confounders, including physician practice patterns, regional access disparities, and evolving standards of care. In resource-constrained settings with limited salvage options, delayed retreatment may inflate TTNT and overestimate efficacy; conversely, rapid access to novel agents can shorten TTNT without altering the underlying disease trajectory [6,15].

In conclusion, TTNT enriches CLL endpoint portfolios by bridging trial efficacy with patient-centered outcomes, but its use requires regimen-specific contextualization and bias-mitigated validation. Future research should prioritize individual patient data (IPD) meta-analyses and standardized retreatment criteria to solidify TTNT’s role in accelerating drug approvals while safeguarding against misestimation of net benefit [29].

## 5. Differential Prognostic Value of MRD Across Contemporary Treatment Strategies

MRD status measured after a time-limited end of therapy course emerged as a strong predictor of both PFS and OS [30]. Early chemoimmunotherapy platforms (FCR) demonstrated that a deepen responses in CLL could potentially prolong PFS [31]. With the advent of ibrutinib, the value of MRD as a surrogate for PFS diminished, since many patients achieved durable partial responses while remaining MRD-positive during continuous ibrutinib therapy [23]. Persistent low-level disease is common after therapy with BTKis and MRD negativity is rarely achieved. In this context, MRD assessment provides limited additional clinical information and is not meaningfully applicable as a response-adapted endpoint [23].

The use of venetoclax, either as a single agent or combined with anti-CD20 antibodies or BTKis, revived MRD’s central role in trials and reinforced its association with PFS [16,17,18,19,20,21].

Substantial international efforts have improved the standardization and reproducibility of MRD assessment in CLL, enabling its integration into venetoclax-based clinical trials [32]. Prospective studies have further evaluated MRD-guided strategies in which treatment duration or discontinuation is adapted according to uMRD status [33,34,35]. Collectively, these data support uMRD as a dynamic biomarker with potential surrogate endpoint properties in appropriately designed time-limited treatment approaches [30].

However, interpreting undetectable MRD (uMRD) as a surrogate for PFS in CLL requires careful assessment [5,7]. In the CAPTIVATE trial based on ibrutinib + venetoclax (I + V) association, uMRD identified a favorable trajectory, with 5.5-year PFS of 71% in the uMRD group versus 47% in the detectable MRD (dMRD) group [16]. In the same area of I + V combinations, the GLOW study similarly demonstrated an MRD-dependent distinction in early PFS, with 2-year PFS of 93% in the uMRD group versus 79.6% in the dMRD group [17]. By contrast, the AMPLIFY trial revealed attenuation of the MRD–PFS relationship in the acalabrutinib–venetoclax (A + V) arm in the intention-to-treat analysis (uMRD at EoT, 45%; 3-year PFS, 76.5%) [18]. These findings were replicated in the I + V arm of the CLL17 trial where uMRD at EoT was 47.2% and 3-year PFS, 79.4%) [19].

An important consideration in assessing the MRD–PFS association is the definition of the PFS time point [36]. In all published trials examining MRD–PFS, the PFS timepoint is defined as the end of therapy (EoT). This choice can pose a critical limitation, because landmark PFS based on EoT may fail to capture therapy-driven shifts in the MRD–PFS relationship that can occur earlier during treatment [37]. Trial-level analyses that use PFS from the time of therapy initiation detect more reliably regimen-specific MRD–PFS associations across studies [7]. A meta-analysis conducted using these criteria found only a modest association between MRD and PFS across BTKi–venetoclax combinations, whereas the association was stronger in trials that combined venetoclax with an anti-CD20 monoclonal antibody [7]. These findings challenge the universal applicability of MRD as a prognostic marker across CLL treatments, given treatment-dependent kinetics and inter-compartmental variability [5,7].

Overall, some caveat persists mainly regarding the MRD thresholds and assessment methods vary. For instances, flow cytometry versus next-generation sequencing (NGS) approaches used in different trials have different sensitivities, and MRD interpretation can differ across laboratories. Standardization is critical for cross-trial comparability. Moreover, no consensus exists on when MRD should be assessed as a surrogate measurement for PFS or OS [32].

Moreover, MRD dynamics is not homogeneous since it may depend on disease biology and the therapeutic modality. With BTKi–venetoclax combinations patients with unmutated IGHV achieve a condition of undetectable MRD earlier than those with mutated IGHV. However, the former patient population is at a higher risk for faster disease re-emergence [17].

In conclusion, MRD has shown promise as a surrogate endpoint for longer-term disease control and may facilitate earlier decision-making in trial design or adaptive studies. However, formal surrogacy validation in CLL—and for each therapy class—is essential before MRD status is used to support registration or to replace longer-term outcomes [5,7].

## 6. QoL and Patient-Reported Outcomes (PROs)

QoL and PROs provide indispensable insight into the lived experience of patients with CLL, capturing dimensions of disease burden and treatment impact that are not reflected in traditional clinician-reported endpoints such as PFS or OS [38,39,40,41,42,43,44,45]. As CLL is often a chronic, relapsing condition with prolonged treatment exposure, the integration of PROs is particularly important to assess the balance between efficacy and tolerability [46]. Regulatory agencies and international working groups, including the International Workshop on CLL (iwCLL) and the European Medicines Agency (EMA), increasingly emphasize the role of QoL as a key component of benefit–risk evaluation [41,44].

Validated instruments commonly used in CLL include the EORTC QLQ-C30, a widely applied cancer-generic tool assessing global health status and multiple functional domains; the disease-specific EORTC QLQ-CLL17 module, which captures symptoms and concerns unique to CLL (e.g., infections, night sweats, lymphadenopathy-related discomfort); and the FACT-Leu questionnaire, which integrates leukemia-specific symptom burden with general well-being [42]. These instruments enable multidimensional assessment across physical functioning, fatigue, pain, emotional and social well-being, and treatment-related adverse effects. Increasingly, digital and electronic PRO (ePRO) platforms are being adopted to improve data completeness and real-time monitoring [42,43].

The timing and frequency of QoL assessment are critical determinants of interpretability. Longitudinal data collection—ideally at baseline, at predefined intervals during therapy, at end of treatment (EoT), and throughout follow-up—is necessary to disentangle short-term treatment-related toxicity from durable improvements in well-being. This is particularly relevant in the context of evolving treatment paradigms in CLL. Fixed-duration regimens, such as venetoclax-based combinations, often show an initial decline in QoL during active therapy followed by meaningful recovery or improvement after treatment discontinuation [40,47]. In contrast, continuous therapies, including BTKis, tend to demonstrate more stable QoL trajectories over time, but these may reflect ongoing low-grade toxicities (e.g., fatigue, diarrhea, cardiovascular effects) that persist with indefinite treatment exposure [48,49].

The analysis and interpretation of QoL data present several methodological challenges. Missing data are a major concern, particularly when associated with disease progression, treatment discontinuation, or death. Such missingness is often not random (informative censoring), leading to potential overestimation of QoL if patients with poorer outcomes are underrepresented in later assessments [50]. Advanced statistical approaches—such as mixed-effects models for repeated measures (MMRM), pattern-mixture models, joint modeling of longitudinal and survival data, and sensitivity analyses—are therefore essential to ensure robust and unbiased conclusions [50]. Additionally, the use of clinically meaningful thresholds, such as minimal important differences (MIDs), is critical for interpreting whether observed changes are not only statistically significant but also relevant to patients [51].

Recent phase III trials underscore the importance of integrating QoL endpoints into CLL research. In the CLL13 trial, venetoclax-based, fixed-duration combinations were associated with improved QoL recovery following treatment cessation, highlighting the value of treatment-free intervals [40]. Similarly, studies such as CLL14 have demonstrated sustained or improved QoL outcomes with time-limited venetoclax regimens compared with chemoimmunotherapy [47]. Conversely, trials evaluating continuous BTKi therapy (e.g., RESONATE-2, ELEVATE-TN) have shown generally stable QoL over time, albeit with ongoing treatment-related symptom burden [48,49]. Notably, QoL trajectories may diverge even when differences in PFS are modest, suggesting that PROs provide complementary and sometimes differentiating information beyond traditional efficacy metrics [41].

Beyond clinical trials, real-world evidence further reinforces the importance of QoL assessment, particularly in older and comorbid populations who may experience greater treatment-related burden. Incorporating PROs into routine clinical practice can also facilitate shared decision-making, allowing treatment selection to align more closely with patient preferences, values, and tolerance for risk [52,53]. In summary, QoL and PROs should not be regarded as secondary or exploratory endpoints but as integral components of clinical evaluation in CLL [41]. Their systematic incorporation into trial design, analysis, and reporting is essential for a comprehensive assessment of net clinical benefit, especially in an era of multiple effective therapeutic options with differing toxicity profiles and treatment durations. Future research should continue to refine PRO methodologies, standardize reporting, and explore the integration of QoL data into composite endpoints and health economic evaluations [41].

## 7. Practical Framework for Selecting Surrogate Endpoints in CLL

To extend the discussion beyond a general summary of known limitations, surrogate endpoint selection in CLL should be treated as a decision problem rather than a single validation problem. The key issue is not whether an endpoint is universally valid, but whether it is fit for a specific therapeutic architecture and a specific clinical or regulatory purpose [54]. This is particularly relevant in CLL because surrogate relationships vary across fixed-duration venetoclax-based regimens and continuous BTKi therapy, and because the same endpoint may carry different meaning depending on whether the goal is registration, comparative effectiveness research, or bedside decision-making (Table 1).

Fixed-duration regimens create a natural end-of-therapy landmark, making MRD biologically meaningful as a marker of depth of remission and treatment-free disease control, whereas continuous BTKi exposure weakens the interpretability of MRD as a general surrogate because patients may remain MRD-positive despite prolonged clinical benefit [41,55]. In contrast, TTNT may be more informative in continuous therapy because it captures both progression and intolerance, which is clinically relevant when discontinuation is driven by adverse events rather than disease relapse [6]. Accordingly, the same endpoint can have very different interpretive value depending on whether therapy is finite or indefinite.

Older and frail patients require an additional layer of interpretation. In these populations, competing mortality, comorbidity burden, and the practical consequences of prolonged treatment can reduce the utility of PFS as a stand-alone measure of benefit [3,55]. For such patients, TTNT, PROs, and toxicity-adjusted composites may better reflect the trade-off between disease control and treatment burden than a progression-based endpoint alone. This is important because a favorable PFS result may still fail to translate into meaningful improvement in daily functioning, symptom burden, or quality of life [6].

Relapsed or refractory disease presents a further challenge because endpoint meaning is shaped by the availability and timing of salvage therapy. In this setting, TTNT may be clinically useful, but it is also vulnerable to confounding by access to subsequent treatments and physician practice patterns, which can shorten or lengthen the interval to retreatment without necessarily changing the underlying disease biology [6]. For that reason, TTNT should not be treated as a simple re-placement for PFS; rather, it should be interpreted as a complementary endpoint that is especially informative when the goal is to understand treatment durability from the patient’s perspective [15]. The recent literature supports this cautious position by emphasizing the need for individual-level and trial-level validation before TTNT is used as a surrogate for regulatory decisions [6].

A multidimensional approach integrating different surrogate endpoints would be particularly relevant in the current treatment landscape, where highly effective subsequent therapies may attenuate OS differences and where delaying disease progression and treatment initiation represents an important clinical objective (Table 2). Of note, although our proposed integrated surrogate endpoint profiles were developed specifically for CLL, they may also have broader applicability to other indolent lymphomas.

## 8. Conclusions

Surrogate endpoints offer a pragmatic means to accelerate the evaluation of new therapies in CLL. However, surrogacy is not a one-size-fits-all proposition; it requires formal validation within the specific patient population, treatment context, and class of interventions under study [15]. In CLL, trials spanning therapeutic modalities—BTKis (ibrutinib, acalabrutinib, zanubrutinib), BCL-2 inhibitors (venetoclax), PI3K inhibitors (idelalisib, duvelisib), and cellular therapies such as chimeric antigen receptor (CAR) T-cell products and bispecific antibodies—engage disease biology and treatment-related toxicity in distinct ways [6].

The methodological toolkit for validating surrogate endpoints in CLL—meta-analytic correlations, trial-level surrogacy models, and causal-inference frameworks—can be challenging [56]. Single-trial signals are rarely sufficient, and robust conclusions require broader, mechanism-aligned evidence across similar regimens plus explicit assessment of transportability and competing risks [30]. Moreover, translating surrogates into practice favors adaptive trial designs with prespecified, validated surrogacy criteria that pair disease-centered surrogates with patient-centered outcomes and standardize assessments to enable cross-trial comparability.

A key development addressed in the 2026 EHA CLL guidelines is the integration of the ESMO-MCBS. This reflects an important step toward a value-based, patient-centered model of care. In a therapeutic landscape characterized by multiple highly effective targeted agents with broadly comparable efficacy but differing toxicity profiles, treatment duration, and monitoring requirements, the ESMO-MCBS offers a structured and transparent framework for evaluating the relative clinical value of competing options beyond traditional efficacy endpoints alone [8].

By translating complex clinical trial outcomes into standardized estimates of magnitude of clinical benefit, the scale enhances shared decision-making and supports more effective patient–physician communication [57]. This facilitates alignment of treatment selection with individual patient preferences, values, and life context, while improving the interpretation of therapeutic trade-offs in routine clinical practice. From a health system perspective, it may also contribute to more equitable and efficient resource allocation by prioritizing interventions that deliver the greatest clinical value.

## Figures and Tables

**Table 1:** Practical framework for selecting surrogate endpoints in chronic lymphocytic leukemia (CLL).

Clinical Setting	Preferred Endpoint(s)	Best Use Case	Why It Is Informative	Key Caveat
Fixed-duration venetoclax-based therapy	MRD, PFS, TTNT	Post-treatment assessment, regimen comparison, treatment-free remission	MRD reflects depth of remission after end of therapy; PFS captures disease control; TTNT captures need for retreatment	MRD performance is regimen-specific and depends on assay standardization and timing
Continuous BTKi therapy	PFS, TTNT, toxicity-adjusted outcomes	Ongoing disease control, tolerability assessment, comparative effectiveness	TTNT captures both progression and discontinuation for intolerance; PFS measures progression during treatment	MRD is less reliable as a universal surrogate in continuous exposure settings
Older or frail patients	TTNT, QoL, PROs, event-based or toxicity-adjusted composites	Net clinical benefit, patient-centered evaluation	Competing mortality and treatment burden make progression-only measures less informative	PFS may overstate benefit if comorbidity and toxicity are ignored
Relapsed or refractory disease	PFS, TTNT, symptom- and toxicity-informed outcomes	Treatment durability, sequencing decisions	Prior therapy exposure and heterogeneous salvage options make time-to-event outcomes clinically relevant	TTNT may be influenced by access to subsequent therapies and physician practice patterns
Regulatory context	PFS, MRD only with class-specific validation, confirmatory OS/QoL	Accelerated approval, labeling decisions	Regulatory decisions require evidence at both patient and trial-level	Surrogacy should not be generalized across therapies or populations without transportability testing

**Table 2:** Examples of integrated surrogate endpoint profiles and their clinical interpretation in CLL.

Integrated Endpoint Profile	Clinical Interpretation	Clinical Applicability
uMRD + prolonged PFS + improved OS + maintained QoL	Strong evidence of durable clinical benefit	Supports high overall treatment effectiveness
uMRD + prolonged PFS + prolonged TTNT + maintained QoL (immature OS)	Deep and durable disease control with delayed need for subsequent therapy	Particularly relevant for evaluating novel and fixed-duration strategies
Improved ORR without sustained uMRD, PFS, or TTNT benefit	Early response without durable disease control	Should be interpreted cautiously and not considered sufficient evidence of long-term benefit
Prolonged PFS + prolonged TTNT + maintained QoL without OS improvement	Meaningful disease control with delayed retreatment and preserved patient well-being	Clinically relevant in settings with effective salvage therapies
Improvement in a single endpoint only	Limited evidence of overall treatment benefit	Requires confirmation with additional clinical and patient-reported outcomes

## Data Availability

The data that support the findings of this study are available from the corresponding author upon reasonable request.
